# Comparison of Different Histological Staining Methods for Detection of Helicobacter pylori Infection in Gastric Biopsy

**DOI:** 10.7759/cureus.27316

**Published:** 2022-07-26

**Authors:** Rita Yadav, Mala Sagar

**Affiliations:** 1 Department of Pathology, Vivekananda Polyclinic and Institute of Medical Sciences, Lucknow, IND; 2 Department of Pathology, King George's Medical University, Lucknow, IND

**Keywords:** gastritis, gimenez stain, gastric biopsies, staining, helicobacter pylori

## Abstract

Background: Helicobacter pylori gastritis affects two-thirds of the world's human population. Among various invasive and non-invasive tests, histology play a very important role in detecting H. pylori in gastric biopsies. In histology, for detection of H. pylori, we use different histological staining techniques like routine haematoxylin & eosin (H&E) stain, Giemsa stain, Gimenez stain, and periodic acid Schiff - Alcian blue (PAS-AB) stain.

Objective: Aim of our study was to evaluate these different histopathological staining techniques for detecting H. pylori in gastric mucosal biopsies and to determine the sensitivity, specificity, positive predictive value, negative predictive value and diagnostic accuracy of H&E stain, Gemenez stain and PAS-AB in the detection of H. pylori in gastric biopsies using Giemsa stain as the reference standard.

Materials and Methods: It was a prospective descriptive study design of 45 gastric biopsies of patients having gastritis. This study was conducted at Vivekananda Polyclinic and Institute of Medical Sciences over a period of one year, from March 2021 to February 2022. From each formalin-fixed paraffin-embedded block, four glass slides were prepared and stained with H&E stain, Giemsa stains, Gemenez stain, and PAS-AB stain to detect the presence/absence of H. pylori in gastric biopsies. Sensitivity, specificity, positive predictive value, negative predictive value and diagnostic accuracy were assessed.

Results: Various staining techniques for detecting H. pylori in gastric mucosal biopsies were compared. In reference to Giemsa stain results, statistical analysis indicates that the diagnostic accuracy of the Gimenez stain, H&E stain, and PAS-AB stain were 95.6%, 91.1%, and 84.4% respectively. Gimenez stain is confirmed to be better than H&E stain and PAS-AB stain to detect H. pylori in 45 gastric biopsies of patients having gastritis. PAS-AB stain is the worst stain to detect H. pylori in gastric biopsies.

Conclusion: Gimenez stain has higher diagnostic accuracy than PAS-AB stain in the detection of H. pylori in gastric biopsy. In fact, Gimenez stain has high sensitivity, specificity and diagnostic accuracy as compared to traditional H&E stain while PAS-AB stain has lower sensitivity, specificity and diagnostic accuracy. Thus, Gimenez stain is also recommended for the detection of H. pylori in gastric biopsy.

## Introduction

Helicobacter pylori is the most common human pathogen and affects various gastroduodenal diseases [1]. It is responsible for the onset of several gastric pathologies ranging from gastritis to gastric carcinoma [2]. It is a well-defined spiral-shaped, gram-negative bacteria [3]. This microorganism was first discovered by Marshall et al. [4]. In 1994, H. pylori was categorized as a class I definite biological carcinogen. In fact gastric carcinogenesis is mainly evoked by the CagA-positive strain of H. pylori [5]. Approximately 80% of the population may be infected by the age of 20 in developing countries like India [6]. H. pylori gastritis has major complications including gastric adenocarcinoma and gastric mucosa-associated lymphoid tissue lymphoma [7]. So H. pylori eradication is a preventative measure for gastric cancer [8]. A wide range of laboratory investigations is available for identification of H. pylori in gastric biopsies [1,2,9-13]. These are both non-invasive and invasive tests [1,9,11]. Non-invasive tests include serology, urea breath test, and stool antigen test. Invasive tests include histology, rapid urease test and culture [1,9]. Histology has excellent sensitivity and specificity, especially with a special stain like Giemsa stain [1,11,12,14-16]. Different histopathological staining methods have been used for many years to detect H. pylori in gastric biopsies, including routine haematoxylin & eosin (H&E) stain and other special stains such as Giemsa, periodic acid Schiff - Alcian blue (PAS-AB), Gimenez, Steiner, Warthin-Starry, Toluidine blue and immune stain [10-17]. This study was designed to evaluate the preferred histological staining methods for detecting H. pylori in gastric biopsies. We performed prospective descriptive study to determine the sensitivity, specificity, positive predictive value, negative predictive value and diagnostic accuracy of H&E stain, Gimenez stain and PAS-AB in the detection of H. pylori in gastric biopsies using Giemsa stain as the reference standard.

## Materials and methods

This was a prospective descriptive study that analysed 45 endoscopic gastric biopsies of patients having gastritis or/and gastric ulcer. This study was conducted in the pathology department of our institution, Vivekananda Polyclinic and Institute of Medical Sciences, over a period of one year, from March 2021 to February 2022. Ethical approval from the institutional ethical committee was taken along with informed consent obtained from each patient (approval number VPIMS/23/2022). Inclusion criterion was the presence of gastritis. Exclusion criteria were cases with dysplasia and carcinoma of stomach. Forty-five formalin-fixed, paraffin-embedded blocks of gastric biopsies were collected. After that four glass slides were prepared from each paraffin-embedded block of gastric biopsies. Then these glass slides were stained with H&E stain, Giemsa stain, Gimenez stain and PAS-AB stain, according to standard protocols. For control of the study two pathologists examined all the sections of H&E stain, Giemsa stain, Gimenez stain, and PAS-AB stain in combination for each case to detect the presence/absence of H. pylori through a microscope under oil immersion field. These slides were examined for the presence of H. pylori mainly in the mucus and gastric pits. After that data were collected in the form of H. pylori-positive gastritis and H. pylori-negative gastritis according to different types of stains. Giemsa stain results were used as reference for the calculation of true positive, true negative, false positive, and false negative values for other histopathological stains (H&E, Gimenez stain, PAS-AB stain). Then we performed statistical analysis in terms of sensitivity, specificity, positive predictive value (PPV), negative predictive value (NPV) and diagnostic accuracy (DA) of the H&E stain, Gimenez stain and PAS-AB stain.

## Results

**Figure 1:**
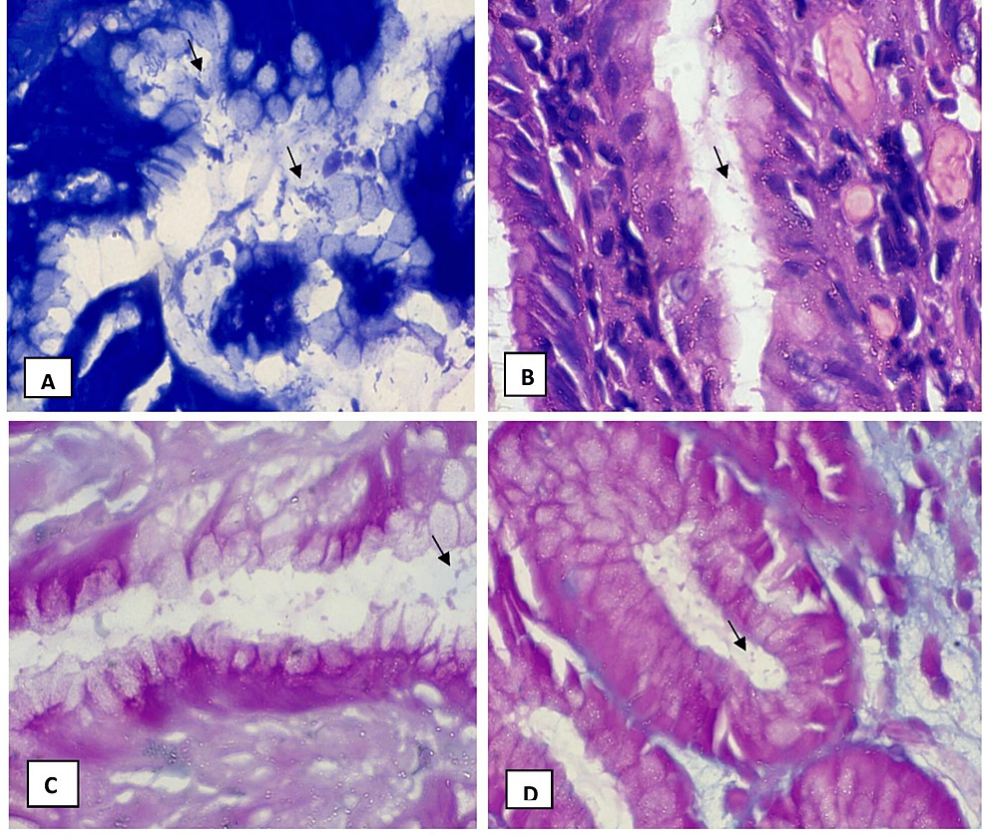
Arrow indicates the spiral-shaped Helicobacter pylori microorganism present in Giemsa stain 400x (A), H&E stain 400x (B), Gimenez stain 400x (C), PAS-AB stain 400x (D) PAS-AB: periodic acid Schiff - Alcian blue

A total of 45 patients who had gastritis or/and gastric ulcer, were participated in this prospective descriptive study. Range of age of patients included in the study was from 19 to 80 years. Twenty-three patients were female and 22 patients were male. Different histological stains are used for the direct detection of H. pylori in gastric biopsies. These stains include H&E stain, Giemsa stain, Gimenez stain and PAS-AB stain. The spiral-shaped H. pylori organism can be directly identified in high magnification with oil immersion field by microscope (Figure 1).

Detection of H. pylori in gastric biopsies by different histological staining methods including Giemsa stain, H&E stain, Gimenez stain and PAS-AB stain are given below in Table 1.

**Table 1:** Detection of Helicobacter pylori in different histological staining sections PAS-AB: periodic acid Schiff - Alcian blue

S.N.	Staining methods	Positive	Negative	Total
1.	Giemsa stain	18	27	45
2.	Haematoxylin & eosin	14	31	45
3.	Gimenez stain	16	29	45
4.	PAS-AB stain	21	24	45

In literature, most of the studies said that Giemsa stain has better sensitivity and specificity for detection of H. pylori in gastric biopsies. In reference to Giemsa stain, all data were statistically analysed in terms of sensitivity, specificity, PPV, NPV, and DA were calculated for comparative analysis which are mentioned in Table 2.

**Table 2:** Comparison of different histological staining methods in terms of sensitivity, specificity, PPV, NPV and DA PPV: positive predictive value, NPV: negative predictive value, DA: diagnostic accuracy, PAS-AB: periodic acid Schiff - Alcian blue

Staining Method	Finding	Giemsa stain		Sensitivity	Specificity	PPV	NPV	DA
		+ve	-ve					
Haematoxylin & eosin	+ve	14	00	77.8	100	100	87.1	91.1
	-ve	04	27					
Gimenez stain	+ve	16	00	88.9	100	100	93.1	95.6
	-ve	02	27					
PAS-AB stain	+ve	18	03	81.8	86.9	85.7	83.3	84.4
	-ve	04	20					

With respect to Giemsa stain, the sensitivity and specificity of H&E were 77.8% and 100% respectively. Further PPV, NPV and DA of the test were 100%, 87.1% and 91.1% respectively.

With respect to Giemsa stain, the sensitivity and specificity of Gimenez stain were 88.9% and 100% respectively. Further PPV, NPV and DA of the test were 100%, 93.1% and 95.6% respectively.

With respect to Giemsa stain, the sensitivity and specificity of PAS-AB stain were 81.8% and 86.9% respectively. Further PPV, NPV and DA of the test were 85.7%, 83.3% and 84.4% respectively.

Gimenez stain was superior to both H&E and PAS-AB stains in detection of H. pylori in gastric biopsies with 95.6% diagnostic accuracy test. It is also obvious that among the three stains (H&E, Gimenez, PAS-AB), PAS-AB stain is the worst stain for detection of H. pylori in gastric biopsies with lower diagnostic accuracy test that is 84.4% in comparison to Gimenez stain that is 95.6%.

## Discussion

Half of the world's human population is infected with H. pylori. It is the most common gastric pathogen that colonizes the mucous lining of the stomach [1]. H. pylori is the only bacterium known to colonize the harsh acidic environment of the human stomach [3]. It produces urease enzyme which is responsible for converting urea into bicarbonate and ammonia, thus elevating the stomach pH [5]. The prevalence of H. pylori in the Indian subcontinent is 80% and most commonly manifests as peptic ulcer disease [6]. H. pylori is the strongest risk factor for the development of gastric cancer [2]. Recent meta-analyses suggest that the relative risk of developing gastric cancer is two to three times higher for people infected with H. pylori than for those without infection [18]. In fact, the CagA positive strain of H. pylori increases the risk of gastric cancer as compared to CagA negative strain of H. pylori [5]. CagA is injected into gastric epithelial cells via type IV secretion system [3]. In the present study, age and gender distribution of H. pylori infection correspond to Agarwal et al. [6].

Detection of H. pylori through various tests includes invasive and non-invasive tests. Invasive tests include histology, rapid urease test (RUT), culture, and polymerase chain reaction (PCR). Non-invasive test include urea breath test (UBT), stool antigen test, and serology [1,9,12]. In literature various studies have compared the sensitivity and specificity of the invasive and non-invasive tests in the diagnosis of H. pylori infection [1,9]. Among various non-invasive tests, UBT is a gold standard in the diagnosis of H. pylori infection in asymptomatic patients, paediatric patients and elderely patients [1,7,9]. Advantage of this test is to avoid endoscopy. UBT is a very popular test for clinicians to confirm bacterial eradication after treatment of patient [9]. Among various invasive tests, histology is an excellent and most commonly used diagnostic method [1,9]. This method has various advantages and disadvantages. The advantages of this method are that (i) It is gold standard for direct visualization of H. pylori (ii) To assess the morphological condition of the gastric mucosa (iii) To diagnose precancerous gastric lesion like atrophic gastritis and intestinal metaplasia (iv) Early detection of gastric carcinoma. The disadvantages of this method are (i) Histology required endoscopic procedure which is an invasive procedure (ii) Contradictory results following PPI consumption (iii) It will not detect H. pylori in cases of low density of bacteria (iv) It is time-consuming and relatively expensive (v) Interobserver variability in assessment [1,2,9,11]. Diagnostic accuracy of histology can be improved by multiple biopsies taken from different sites of gastric mucosa. According to updated Sydney system, biopsy should be taken from five different sites of gastric mucosa for optimal assessment of both gastritis and H. pylori status [1]. If multiple biopsies are not possible, at least two gastric mucosal biopsies (each from the antrum and corpus) are taken [19].

Several histopathological staining panels have been used for detection of H. pylori in gastric biopsies [13]. H&E stain is routinely used in almost all laboratories but diagnostic yield of histology is improved with the help of special stains like Giemsa, Gimenez, Steiner, Warthin Starry, PAS-AB, Toluidine blue and immune stain [10-13]. According to literature Giemsa stain has excellent sensitivity and specificity in the detection of H. pylori infection in gastric biopsies [11,12,14]. For detection of H. pylori correct and prompt diagnosis is essential. Therefore most laboratories use an additional staining method along with H&E stain in the identification of the organism [14-16]. Giemsa stain is simple, rapid, inexpensive and provides consistent results [12,14]. So this stain is more commonly used as an additional stain [1,12]. Giemsa stain is also a better diagnostic method for detection of H. pylori infection as compared to rapid urease test [12]. So in our study Giemsa stain results were considered as standard reference to measure the performance of other stains in terms of sensitivity, specificity, PPV, NPV and DA.

The data from this study showed that diagnostic accuracy of the Gimenez stain was higher as compared to H&E stain (Table 2) which means the ability of this stain to correctly identify microorganisms in gastric biopsies. This finding is consistent with findings of Sied et al. [15]. In our study, PAS-AB stain is the worst choice to detect H. pylori in gastric biopsies. This finding is consistent with findings of Alkhamiss et al. [11]. The accuracy of histology depends on various factors like pathologists’ experience, staining technique, and low or high quantity of H. pylori colonization in the gastric mucosa [1,9].

Sensitivity and specificity of H&E stain, Gimenez stain and PAS-AB stain in the detection of H. pylori are shown in Table 2. From this Table it was clear that Gimenez stain has high sensitivity (88.9%) and high specificity (100%) as compared to the other two stains (H&E and PAS-AB). This finding corresponds to Sied et al. [15]. According to our study, PAS-AB stain has lower specificity as compared to H&E and Gimenez stain because of high false positivity as well as false negativity. This occurs due to dirty background of the slide. So, among all stains under study, PAS-AB was the worst stain for diagnosis of H. pylori. This finding corresponds to the finding of Alkhamiss et al. [11].

PPV is the proportion of patients with a positive test who have the disease, while NPV is the proportion of patients with a negative test who do not have the disease [20]. In our study, PPV and NPV of H&E stain and Gimenez stain were higher than PPV and NPV of PAS-AB stain.

## Conclusions

According to our study, Gimenez stain has high diagnostic accuracy than H&E and PAS-AB stain in the detection of H. pylori in gastric biopsies. Gimenez stain has high sensitivity, specificity as compared to traditional H&E stain while PAS-AB stain has lower sensitivity, specificity and diagnostic accuracy This study strongly recommends Gimenez stain and Giemsa stain used as additional special stain along with H&E stain for detection of H. pylori in gastric biopsies.
